# An enhanced three-stage design with trend analysis for allergen immunotherapy trials

**DOI:** 10.1371/journal.pone.0291533

**Published:** 2023-09-14

**Authors:** Xinyu Tang, Ronald L. Rabin, Lihan K. Yan

**Affiliations:** 1 Office of Biostatistics and Pharmacovigilance, Center for Biologics Evaluation and Research (CBER), U.S. Food and Drug Administration (FDA), Silver Spring, MD, United States of America; 2 Office of Vaccines Research and Review, CBER, FDA, Silver Spring, MD, United States of America; Sreenidhi Institute of Science and Technology, INDIA

## Abstract

We previously introduced a three-stage design and associated end-of-stage analyses for allergen immunotherapy (AIT) trials. End-of-stage differences alone may not provide a fuller picture of Stages 2 and 3 effects because they may depend upon stage-specific durations. Therefore, we introduce an additional trend analysis to evaluate the difference in progression curves of two groups over the entire stage. Results from such analysis are used to inform persistence of end-of-stage benefit and thus provide evidence for stagewise effects beyond the study periods. We jointly apply end-of-stage and trend analyses to support the enhanced three-stage design to determine treatment response over time and sustained response to AIT. A simulation study was performed to illustrate the statistical properties (bias and power) of trend analyses under varying statistical missing mechanisms and effect sizes. The extent of bias depended on the missing mechanism and magnitude. Powers were largely driven by effect and sample sizes as well as pre-specified success margins, particularly of relative trend. As an illustration, assuming relative treatment differences of 25–30%, stagewise dropout rate of 15%, and parallel outcome progressions, a sample size of 200 per group may achieve 97% power to demonstrate a treatment effect and 53% power to demonstrate a sustained effect post-treatment. Trend analysis supplements the end-of-stage analysis to enhance the statistical claims of stagewise effects. Inferential statistics support our proposed trend analysis for evaluating benefits of AIT over time and inform clinical understanding and decisions.

## Introduction

We previously introduced a three-stage design for allergen immunotherapy (AIT) trials to characterize the effects of an AIT product through stagewise statistical evaluations [1]. Briefly, Stage 1 is a double-blind placebo-controlled randomized (DBPCR) study to evaluate an initial treatment effect in participants who received the active treatment compared to those who received placebo. If an initial treatment effect is demonstrated by the end of Stage 1, the trial will continue to Stage 2. In Stage 2, participants in the treatment group in Stage 1 continue to receive treatment (referred to as original treatment group hereafter), while the initial placebo recipients cross over to receive the treatment (referred to as crossover group hereafter) in an open-label fashion. Upon completion of Stage 2, the trial will continue to Stage 3. Stage 3 is a post-treatment follow-up study, in which all participants are followed for a pre-determined length of time to evaluate whether the treatment effect is maintained after treatment is discontinued. Such design preserves the initial randomization which allows further evaluations of the magnitude and duration of the treatment responses at later stages while provides timely access to active therapy for placebo recipients which potentially reduces dropouts due to perceiving little or no immediate benefit as well as differential dropouts for reasons related to treatment in later stages.

**Fig 1:**
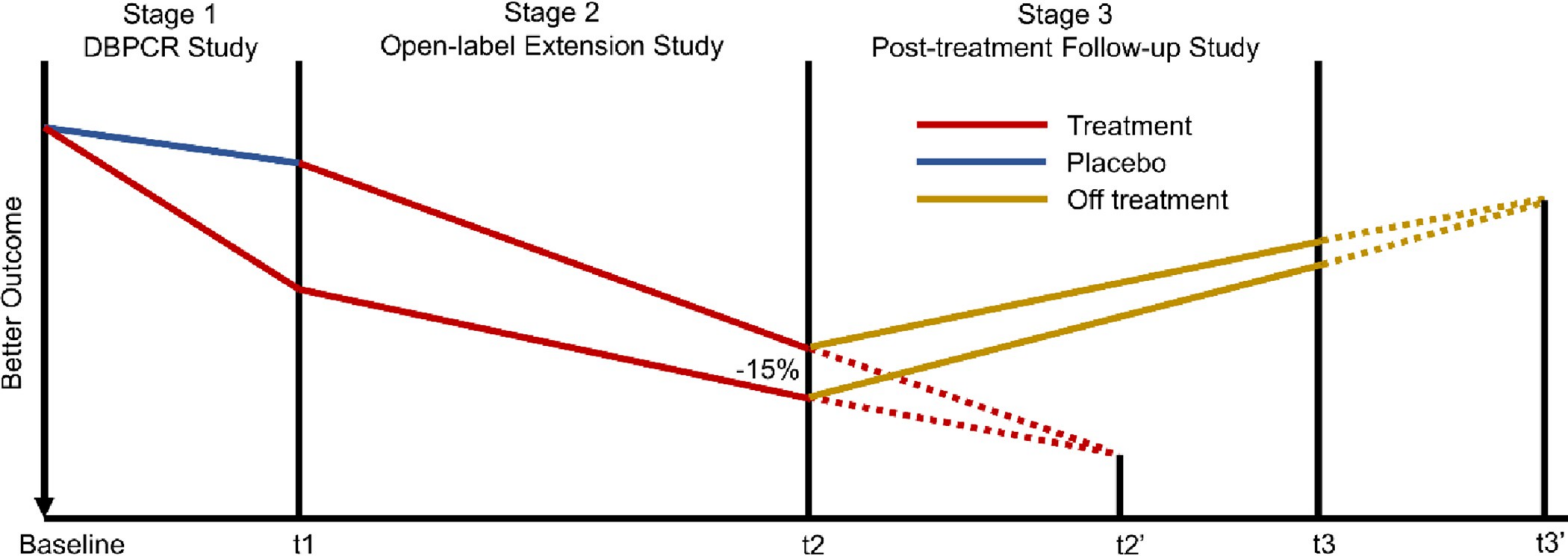
A hypothetical three-stage trial.

Currently, most AIT trials with long-term follow-up have focused on descriptive statistics. However, as described in Tang et al. (2021) [1], following conventional analysis strategy, comparative analysis of data collected at the end of each stage was proposed to evaluate the stagewise effects. These end-of-stage analyses can provide information on an initial treatment effect (Stage 1 effect), an effect associated with time of introduction and length of treatment (Stage 2 effect), and a potential sustained effect after treatment is withdrawn (Stage 3 effect). However, end-of-stage analyses are subject to stage-specific durations. In other words, end-of-stage differences may be impacted by the duration of the underlying stage. For example, in a hypothetical three-stage trial (Fig 1), an initial treatment effect is demonstrated by the end of Stage 1. The trial continues to Stage 2. At the end of Stage 2 (time point t2), the difference between two groups meets the pre-specified success margin of -15%, and thus a Stage 2 effect is claimed. However, if we extend the duration of Stage 2 from time point t2 to t2’, following the same trend the difference may diminish at time point t2’, and thus a Stage 2 effect will not be claimed. Similarly, after the treatment is withdrawn, the difference observed at the end of Stage 3 (time point t3) could also diminish when the two progression lines converge at time point t3’, an extended duration time point from t3. Therefore, the lengths of the stages are important aspects in consideration when we describe and interpret the comparative results in the three-stage design.

Since the lengths of stages, particularly for Stages 2 and 3, are often constrained due to budget and operational consideration, analysis of the outcome progression would provide insights for expected stage effects beyond the underlying study periods. In the literature, comparing the progression of outcome between two groups has been suggested to infer persistence of a treatment effect for diseases that progress slowly such as Parkinson’s disease and Alzheimer’s disease [2–4]. Therefore, given the circumstances when comparative results from end-of-stage analyses alone do not fully describe the stagewise effects, we further introduce trend analyses, in which the progression curves of the two groups over the entire Stage 2 (or 3) are compared and evaluated additionally. These complementary analyses would provide further information for understanding the profile and clinical benefit of an AIT and assist decision-making in clinical practice.

The remainder of this paper is organized as follows. The “Materials and Methods” section describes the notations and statistical hypotheses for Stages 2 and 3 trend analyses, the statistical methods for estimating the trend differences, the relationship between end-of-stage differences, trend differences and test margins, and the set-up of the simulation study to evaluate the performance of the proposed trend analyses under various levels of Stage 2 or 3 effect and missing mechanisms. The “Results” section summarizes the simulation results in terms of estimates, type I error rates, and powers for Stages 2 and 3 trend analyses. The “Discussion” section summarizes the contributions of the trend analyses in aiding interpretation of the Stages 2 and 3 results and describes the caveats of the proposed methods and future research directions.

## Materials and methods

### Trend analysis

Because progression can be best described when study outcome is periodically assessed, we focus on AIT trials where repeatedly measured outcomes are available. We follow the same notations used in Tang et al. (2021) [1]. For Stage *i* = 1, 2, 3, *X*_*ti*_ and *X*_*pi*_ are end-of-stage *i* responses for the original treatment and crossover groups respectively, and *S*_*i*_ is end-of-stage difference measured by absolute difference (*X*_*ti*_−*X*_*pi*_)or relative difference $\left[100\times \left(X_{ti}-X_{pi}\right)/X_{pi}\right]$. In addition, assuming a linear response curve, we denote *β*_*ti*_ and *β*_*pi*_ the slopes of the original treatment and crossover groups in Stages *i* = 2 and 3, respectively. The between-group trend differences during Stages 2 and 3 (denoted *R*_2_ and *R*_3_) are then measured by the slope ratios *β*_*t*2_/*β*_*p*2_, and *β*_*t*3_/*β*_*p*3_, respectively.

Table 1 shows the proposed stagewise statistical hypotheses for three-stage trials enhanced from Tang et al. (2021) [1]. Hypotheses 1, 2a, and 3a are formulated for end-of-stage analyses to test for differences at the end of Stages 1, 2, and 3 (*S*_1_, *S*_2_ and*S*_3_), respectively. In addition, the response curves over Stages 2 and 3 are evaluated in trend analyses through the noninferiority comparisons described in Hypotheses 2b and 3b. During Stage 2, we expect to see improving trends (i.e., upward trend if higher outcome is better or downward trend if higher outcome is worse) in both groups as AIT is given, noninferiority of the slope suggests that the rate of improvement in the original treatment group is no less than that in the crossover group. Therefore, a lower bound margin is used in the hypothesis test. On the other hand, during Stage 3, we expect diminishing effect trends (i.e., downward trend if higher outcome is better or upward trend if higher outcome is worse) in both groups as AIT is withdrawn. Noninferiority of the slope suggests that the rate of diminishing effect in the original treatment group is no more than that in the crossover group. Therefore, an upper bound margin is used in the hypothesis test.

**Table 1:** Stagewise statistical hypotheses for an enhanced three-stage design.

Stage	Analysis	Statistical hypothesis	Level of treatment effect evaluated
1 (DBPCR Study)	End-of-Stage 1 analysis	Hypothesis 1: H_0_: *S*_1_≤(≥)*δ*_1_ vs. H_a_: *S*_1_>(<)*δ*_1_ when higher outcome is better (worse)	Stage 1 effect
2 (Open-label Extension Study)	End-of-Stage 2 analysis	Hypothesis 2a: H_0_: *S*_2_≤(≥)*δ*_2_ vs. H_a_: *S*_2_>(<)*δ*_2_ when higher outcome is better (worse)	Stage 2 effect
	Stage 2 trend analysis	Hypothesis 2b: H_0_: *R*_2_≤*λ*_2_ vs. H_a_: *R*_2_>*λ*_2_	
3 (Post-treatment Follow-up Study)	End-of-Stage 3 analysis	Hypothesis 3a: H_0_: *S*_3_≤(≥)*δ*_3_ vs. H_a_: *S*_3_>(<)*δ*_3_ when higher outcome is better (worse)	Stage 3 effect
	Stage 3 trend analysis	Hypothesis 3b: H_0_: *R*_3_≥*λ*_3_ vs. H_a_: *R*_3_<*λ*_3_	

*S*_1_, *S*_2_ and *S*_3_ indicate end-of-stage 1, 2 and 3 differences, respectively.

*δ*_1_, *δ*_2_, and *δ*_3_ are success margins for end-of-stage 1, 2, and 3 differences, respectively.

*R*_2_, and *R*_3_ indicate Stage 2 and 3 trend differences, respectively.

*λ*_2_, and *λ*_3_ are noninferiority margins for Stage 2 and 3 trend differences, respectively.

For example, if there exists a constant distance between the two progression curves, this will indicate that the end-of-stage benefit will persist, and particularly not diminishing, regardless of the duration of the stage. Therefore, with the incorporation of trend analysis in Stages 2 and 3, statistical claims of Stage 2 or 3 effect would reflect hints on the persistence of difference between two groups beyond the underlying study period.

### Trend estimation

Estimation of the end-of-stage differences *S*_1_, *S*_2_ and *S*_3_ are described in Tang et al. (2021) [1]. We focus on estimation of the Stage 2 and 3 trend differences *R*_2_ and *R*_3_ in this paper. We take estimation of the Stage 2 trend difference *R*_2_ as an example. Let *N* and *K* denote the number of participants and time points available for Stage 2 trend analysis. If we observe the repeatedly measured outcome *x*_*jk*_ for subject *j* at time point *k* during Stage 2 where *j* = 1,…,*N*, and *k* = 1,…,*K*, assuming a linear trend, a mixed model can be fitted to the repeatedly measured outcome during Stage 2 as follows:

$$X_{jk}=b_{0}+b_{1}Group+b_{2}Time+b_{3}Group\times Time+u_{j}+\varepsilon_{jk}$$ (1)

where Group is a dummy variable indicating the group assignment for subject *j* (Group = 1 for the original treatment group and 0 for the crossover group), Time is a continuous variable representing the time point at which the outcome *x*_*jk*_ is measured, *u*_*j*_ is a random effect within subject *j*, and *ε*_*jk*_ is the error term, for *j* = 1,…,*N*, and *k* = 1,…,*K*.

After fitting model (1), we obtain the coefficient estimates ${\hat{b}}_{0},\;{\hat{b}}_{1},\;{\hat{b}}_{2}$, and ${\hat{b}}_{3}$, and the estimated variance-covariance matrix

$$\hat{\Sigma }=[\begin{array}{cccc} {\hat{\sigma }}_{0}^{2} & {\hat{\sigma }}_{01} & {\hat{\sigma }}_{02} & {\hat{\sigma }}_{03}\\ {\hat{\sigma }}_{01} & {\hat{\sigma }}_{1}^{2} & {\hat{\sigma }}_{12} & {\hat{\sigma }}_{13}\\ {\hat{\sigma }}_{02} & {\hat{\sigma }}_{12} & {\hat{\sigma }}_{2}^{2} & {\hat{\sigma }}_{23}\\ {\hat{\sigma }}_{03} & {\hat{\sigma }}_{13} & {\hat{\sigma }}_{23} & {\hat{\sigma }}_{3}^{2} \end{array}],$$

where ${\hat{\sigma }}_{p}^{2}$ is the estimated variance for ${\hat{b}}_{p}$ and ${\hat{\sigma }}_{pq}$ is the estimated covariance between ${\hat{b}}_{p}$ and ${\hat{b}}_{q}\;(p,q=0,1,2,3)$. The slopes of the original treatment and crossover groups during Stage 2 are estimated as ${\hat{\beta }}_{t2}={\hat{b}}_{2}+{\hat{b}}_{3}$, and ${\hat{\beta }}_{p2}={\hat{b}}_{2}$, respectively. The trend difference *R*_2_ is then estimated as the slope ratio ${\hat{\beta }}_{t2}/{\hat{\beta }}_{p2}=\left({\hat{b}}_{2}+{\hat{b}}_{3}\right)/{\hat{b}}_{2}=1+{\hat{b}}_{3}/{\hat{b}}_{2}$. The standard error of the estimated trend difference can be computed using the delta method [5] as

$$SE({\hat{\beta }}_{t2}/{\hat{\beta }}_{p2})=\sqrt{{\hat{\sigma }}_{3}^{2}/{\hat{b}}_{2}^{2}-2{\hat{b}}_{3}{\hat{\sigma }}_{23}/{\hat{b}}_{2}^{3}+{\hat{b}}_{3}^{2}{\hat{\sigma }}_{2}^{2}/{\hat{b}}_{2}^{4}}.$$

The 100(1-α)% confidence interval (CI) around the estimated Stage 2 trend difference is constructed as $\left[{\hat{\beta }}_{t2}/{\hat{\beta }}_{p2}-z_{\alpha /2}SE({\hat{\beta }}_{t2}/{\hat{\beta }}_{p2}),{\hat{\beta }}_{t2}/{\hat{\beta }}_{p2}+z_{\alpha /2}SE({\hat{\beta }}_{t2}/{\hat{\beta }}_{p2})\right]$, where *z*_*α*/2_ is the upper α/2-quantile for the standard normal distribution. The null hypothesis 2b is rejected if the lower limit (LL) of the 100(1-α)% CI around the estimated Stage 2 trend difference is greater than *λ*_2_.

Estimation of the Stage 3 trend difference *R*_3_ and its standard error follows the same statistical approaches as described above. Similarly, the null hypothesis 3b is rejected if the upper limit (UL) of the 100(1-α)% CI around the estimated Stage 3 trend difference is less than *λ*_3_.

### Relationship between end-of-stage differences, trend differences and test margins

In this section, we attempt to use Stage 2 analysis as an example to show intertwined relationship between end-of-stage differences, trend differences and associated test margins following the notations introduced in the “Trend analysis” subsection.

Under linear trends, the Stage 2 trend difference is measured by slope ratio (*R*_2_). When end-of-stage 1 and 2 differences (*S*_1_ and *S*_2_) are measured by absolute difference, the relationship between *S*_1_, *S*_2_, and *R*_2_ is shown below:

$$R_{2}=1+\frac{S_{2}-S_{1}}{\beta_{p2}t_{s2}},$$ (2)

where *t*_*s*2_ indicates the duration of Stage 2.

Alternatively, when end-of-stage 1 and 2 differences are measured by relative difference, the relationship is as follows:

$$R_{2}=1+S_{2}+\frac{X_{p1}}{\beta_{p2}t_{s2}}(S_{2}-S_{1}).$$ (3)

Based on Eqs (2) and (3), the Stage 2 trend difference is related to several components: end-of-stage 1 difference (*S*_1_), comparator Stage 2 slope (i.e., slope of the crossover group during Stage 2), duration of Stage 2, and most importantly end-of-stage 2 difference (*S*_2_). As a result, when end-of-stage 1 data are available and if linear progression is a plausible assumption, there exists a linear relationship between the two quantitative metrics for measuring a Stage 2 effect (*S*_2_ and *R*_2_), given the comparator slope and duration of Stage 2.

The relationships between two effects (*S*_2_ and *R*_2_) shown above can be further rendered in the relationship between two test margins (*δ*_2_ and *λ*_2_) which are the fixed thresholds representing clinically meaningful differences. When higher outcome is worse, as shown in Table 1, the two success criteria 2a and 2b for claiming a Stage 2 effect are *S*_2_<*δ*_2_ and *R*_2_>*λ*_2_, respectively. We expect to see downward trends for an improving outcome as AIT is given for both groups during Stage 2, which accord with negative slopes during Stage 2 (i.e., *β*_*p*2_<0). Incorporating *S*_2_<*δ*_2_ into Eq (2) or (3), success criterion 2a becomes

$R_{2}=1+\frac{S_{2}-S_{1}}{\beta_{p2}t_{s2}}>1+\frac{\delta_{2}-S_{1}}{\beta_{p2}t_{s2}}$, or

$R_{2}=1+S_{2}+\frac{X_{p1}}{\beta_{p2}t_{s2}}(S_{2}-S_{1})>1+\delta_{2}+\frac{X_{p1}}{\beta_{p2}t_{s2}}(\delta_{2}-S_{1})$, respectively.

Therefore, depending on whether $\left[1+\left(\delta_{2}-S_{1}\right)/\beta_{p2}t_{s2}\right]$ or $\left[1+\delta_{2}+X_{p1}(\delta_{2}-S_{1})/\beta_{p2}t_{s2}\right]$ is greater (or less) than *λ*_2_, the success criterion 2a (or 2b) would drive the sample size/power, respectively. When higher outcome is better, the relationship is similar but in a reversed way.

In the following, we present a numerical illustration for the relationship between *S*_2_ and *R*_2_ as shown in Eq (3). Suppose the outcome at the end of Stage 1 was 6.0 and 7.5 for the original treatment and crossover groups, respectively, which corresponds to an end-of-stage 1 relative difference of -20% [*S*_2_ = (6.0–7.5)/7.5 = -20%]. The duration of Stage 2 is 24 months (i.e., *t*_*s*2_ = 24). The slope of the crossover group is -0.15625 during stage 2 (i.e., *β*_*p*2_ = -0.15625). If we choose a test margin of -20% for *S*_2_ and 80% for *R*_2_, namely, *δ*_2_ = -20%, and *λ*_2_ = 80%, then success criterion 2a becomes

$$R_{2}>1+\delta_{2}+\frac{X_{p1}}{\beta_{p2}t_{s2}}(\delta_{2}-S_{1})=1-0.2+\frac{7.5}{(-0.15625)\times 24}(-0.2+0.2)=80\%,$$

which is equivalent to success criterion 2b (*R*_2_>*λ*_2_ = 80%). Therefore, both success criteria 2a and 2b affect sample size/power similarly. In another setting, if we choose a test margin of -15% for *S*_2_ and 80% for *R*_2_, namely, *δ*_2_ = -15%, and *λ*_2_ = 80%, then success criterion 2a becomes

$$R_{2}>1+\delta_{2}+\frac{X_{p1}}{\beta_{p2}t_{s2}}(\delta_{2}-S_{1})=1-0.15+\frac{7.5}{(-0.15625)\times 24}(-0.15+0.2)=65\%,$$

which is less stringent compared to success criterion 2b (*R*_2_>*λ*_2_ = 80%). Hence, in this case, sample size/power would be dominated by success criterion 2b.

The above discussions can be similarly applied to the respective parameters in Stage 3. The purpose of this section is to provide theoretical background for explaining some of the findings described in later sections of the paper. As another overarching purpose, they may also be used as a reference in determining clinically meaningful margin for the treatment difference at the end of the trial given the understanding of the progression profiles of the two groups, or vice versa.

### Trial simulations

**Fig 2:**
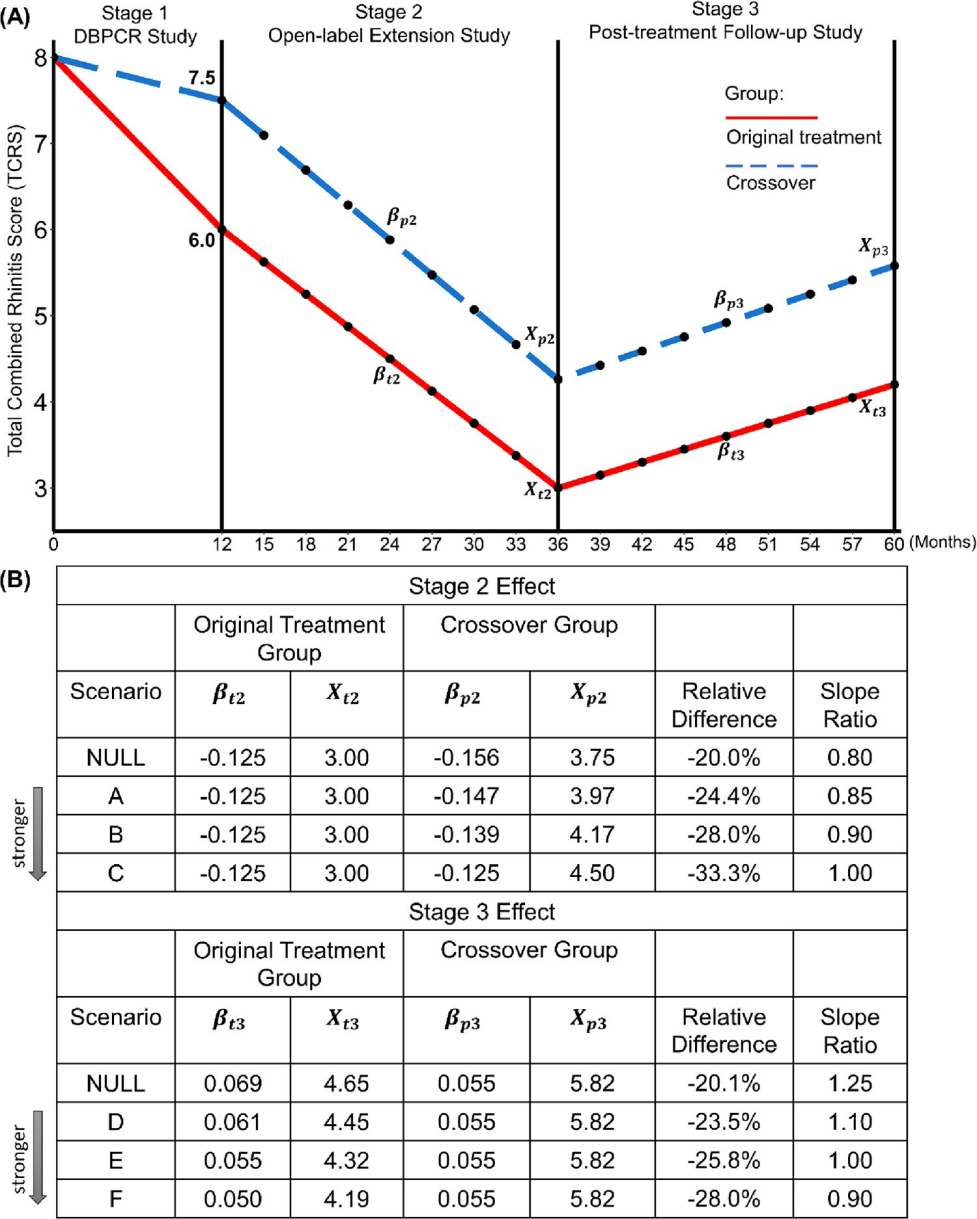
(A) Study durations and frequencies of measured outcomes of the simulated trial; (B) Eight scenarios of different levels of Stage 2 or 3 effect. *X*_*t*2_ = Average TCRS of the original treatment group at the end of Stages 2. *X*_*p*2_ = Average TCRS of the crossover group at the end of Stages 2. *X*_*t*3_ = Average TCRS of the original treatment group at the end of Stages 3. *X*_*p*3_ = Average TCRS of the crossover group at the end of Stages 3. *β*_*t*2_ = Slope of the original treatment group during Stage 2. *β*_*p*2_ = Slope of the crossover group during Stage 2. *β*_*t*3_ = Slope of the original treatment group during Stage 3. *β*_*p3*_ = Slope of the crossover group during Stage 3. Note: All Stage 3 scenarios were investigated following Stage 2 scenario C.

A simulation study was carried out to illustrate testing procedures at different stages and to evaluate the performance of the proposed trend analyses under various levels of Stage 2 or 3 effect and missing mechanisms [missing at random (MAR) or missing not at random (MNAR)]. We employed the same three-stage trial to evaluate the benefits of an AIT in reducing the total combined rhinitis score (TCRS) at 1-, 3-, and 5-years as described in Tang et al. (2021) [1]. Fig 2(A) shows study durations and frequencies of measured outcomes of the simulated trial. Specifically, study durations for Stages 1 through 3 were 12, 24, and 24 months, respectively. Average TCRS measurements were repeatedly obtained every three months starting from Stage 2.

End-of-stage and trend differences were measured by relative difference and slope ratio, respectively. The success margin for end-of-stage differences was chosen to be -15% (i.e., *δ*_1_ = *δ*_2_ = *δ*_3_ = -15%). The non-inferiority margin was chosen to be 80% and 125% for Stages 2 and 3 trend differences (i.e., *λ*_2_ = 80% and *λ*_3_ = 125%), respectively. Under such settings, the success criteria 2a and 2b (corresponding to hypotheses 2a and 2b) for declaring a Stage 2 effect were that the UL of the two-sided 95% CI around the relative difference at the end of Stage 2 was less than -15%, and the LL of the two-sided 95% CI around the slope ratio for Stage 2 was greater than 80%, respectively. Similarly, the success criteria 3a and 3b for declaring a Stage 3 effect were that the UL of the two-sided 95% CI around relative difference at the end of Stage 3 was less than -15%, and the UL of the two-sided 95% CI around the slope ratio for Stage 3 was less than 125%, respectively.

As shown in Fig 2(A). the average TCRS was 8.0 at baseline and reduced to 6.0 and 7.5 at the end of Stage 1 for the original treatment and crossover groups, respectively. Thus, in this simulation, an administration of AIT for the first 12 months was associated with an improvement of 20% relative reduction in TCRS [i.e., (6.0–7.5)/7.5 = -20%]. Fig 2(B) shows eight scenarios of different levels of Stage 2 or 3 effect. For Stage 2 scenarios, the slope of the original treatment group was fixed at -0.125. We then assigned a slope ratio of 0.80 (= noninferiority margin for Stage 2), 0.85, 0.90, and 1.00 for Stage 2 scenario NULL, A, B and C, respectively, demonstrating an increasingly stronger Stage 2 effect. Similarly, the slope of the crossover group was fixed at 0.055 for Stage 3 scenarios. We then assigned a slope ratio of 1.25 (= noninferiority margin for Stage 3), 1.1, 1.0 and 0.9 for Stage 3 scenarios NULL, D, E, and F, respectively, demonstrating an increasingly stronger Stage 3 effect. For illustration purposes, Stage 3 scenarios were investigated following Stage 2 scenario C, and thus the initial score at the beginning of Stage 3 was 3.00 and 4.50 for the original treatment and crossover groups, respectively. As explained in the previous subsection, under the current settings, Stage 2/3 success would be dominated by success criterion 2b/3b under all scenarios.

Under each of the underlying scenarios, 10,000 trials were simulated for a sample size of 200 per group. Data were generated following the same methods as described in Tang et al. (2021) [1]. Model (1) was fitted to estimate the relative difference and slope ratio for each stage. First-order autoregressive correlation structure was introduced when fitting the model to account for longitudinal measurements from the same individual. Trend differences were estimated as described in the “Trend estimation” subsection. Analyses were conducted on both the observed data and ten sets of multiply imputed data from multivariate imputations by chained equations. Estimates from analysis of each set of multiply imputed data were combined following Rubin’s rules.

## Results

With a sample size of 200 per group, there was approximately 97% power to demonstrate Stage 1 effect (relative symptom score reduction is more than 15%) if the true improvement is 20% relative reduction as shown in Fig 2(A).

Estimates and absolute biases of the relative difference and slope ratio under various Stage 2 or 3 effect are shown in Table 2. Both Stages 2 and 3 estimates were generally unbiased under MAR. Under MAR, analyses of imputed data resulted in slightly larger absolute biases compared to those from analyses of observed data. The absolute biases obtained under MNAR were noticeably larger compared to those obtained under MAR. Under MNAR, Stage 3 estimates tended to be more biased as more participants dropped out, with larger absolute biases compared to those of Stage 2 estimates.

**Table 2:** Estimates and absolute biases of the relative differences and slope ratio under Stages 2 and 3 scenarios.

		Stage 2 Effect			
		MAR		MNAR	
Scenario	Parameter	Analysis of Observed Data	Analysis of Imputed Data	Analysis of Observed Data	Analysis of Imputed Data
NULL	Relative Difference (absolute bias)	-19.9% (0.13%)	-19.1% (0.89%)	-19.7% (0.33%)	-19% (1.05%)
	Slope Ratio (absolute bias)	0.799 (0.0009)	0.795 (0.0046)	0.798 (0.0024)	0.794 (0.0060)
A	Relative Difference (absolute bias)	-24.3% (0.14%)	-23.5% (0.98%)	-24.1% (0.34%)	-23.3% (1.12%)
	Slope Ratio (absolute bias)	0.849 (0.0010)	0.843 (0.0066)	0.846 (0.0041)	0.841 (0.0094)
B	Relative Difference(absolute bias)	-27.8% (0.17%)	-26.9% (1.06%)	-27.7% (0.30%)	-26.9% (1.12%)
	Slope Ratio (absolute bias)	0.899 (0.0013)	0.891 (0.0089)	0.895 (0.0050)	0.888 (0.0123)
C	Relative Difference (absolute bias)	-33.2% (0.12%)	-32.2% (1.09%)	-33.1% (0.22%)	-32.2% (1.09%)
	Slope Ratio (absolute bias)	0.999 (0.0008)	0.988 (0.0124)	0.994 (0.0062)	0.982 (0.0175)
		Stage 3 Effect			
		MAR		MNAR	
Scenario	Parameter	Analysis of Observed Data	Analysis of Imputed Data	Analysis of Observed Data	Analysis of Imputed Data
NULL	Relative Difference (absolute bias)	-20.1% (0.02%)	-19.5% (0.60%)	-19.5% (0.62%)	-18.9% (1.18%)
	Slope Ratio (absolute bias)	1.255 (0.0048)	1.244 (0.0058)	1.306 (0.0565)	1.296 (0.0459)
D	Relative Difference (absolute bias)	-23.5% (0.02%)	-22.9% (0.59%)	-23% (0.53%)	-22.4% (1.13%)
	Slope Ratio (absolute bias)	1.101 (0.0007)	1.09 (0.0097)	1.142 (0.0423)	1.134 (0.0341)
E	Relative Difference (absolute bias)	-25.8% (0.01%)	-25.1% (0.64%)	-25.3% (0.52%)	-24.6% (1.14%)
	Slope Ratio (absolute bias)	1.003 (0.0032)	0.993 (0.0070)	1.038 (0.0377)	1.031 (0.0306)
F	Relative Difference (absolute bias)	-28% (0.01%)	-27.4% (0.66%)	-27.6% (0.48%)	-26.9% (1.10%)
	Slope Ratio (absolute bias)	0.902 (0.0018)	0.892 (0.0081)	0.929 (0.0292)	0.922 (0.0222)

MAR = missing at random; MNAR = missing not at random

Note: Refer to Fig 2(B) for the various effects under Stages 2 and 3 scenarios.

**Fig 3:**
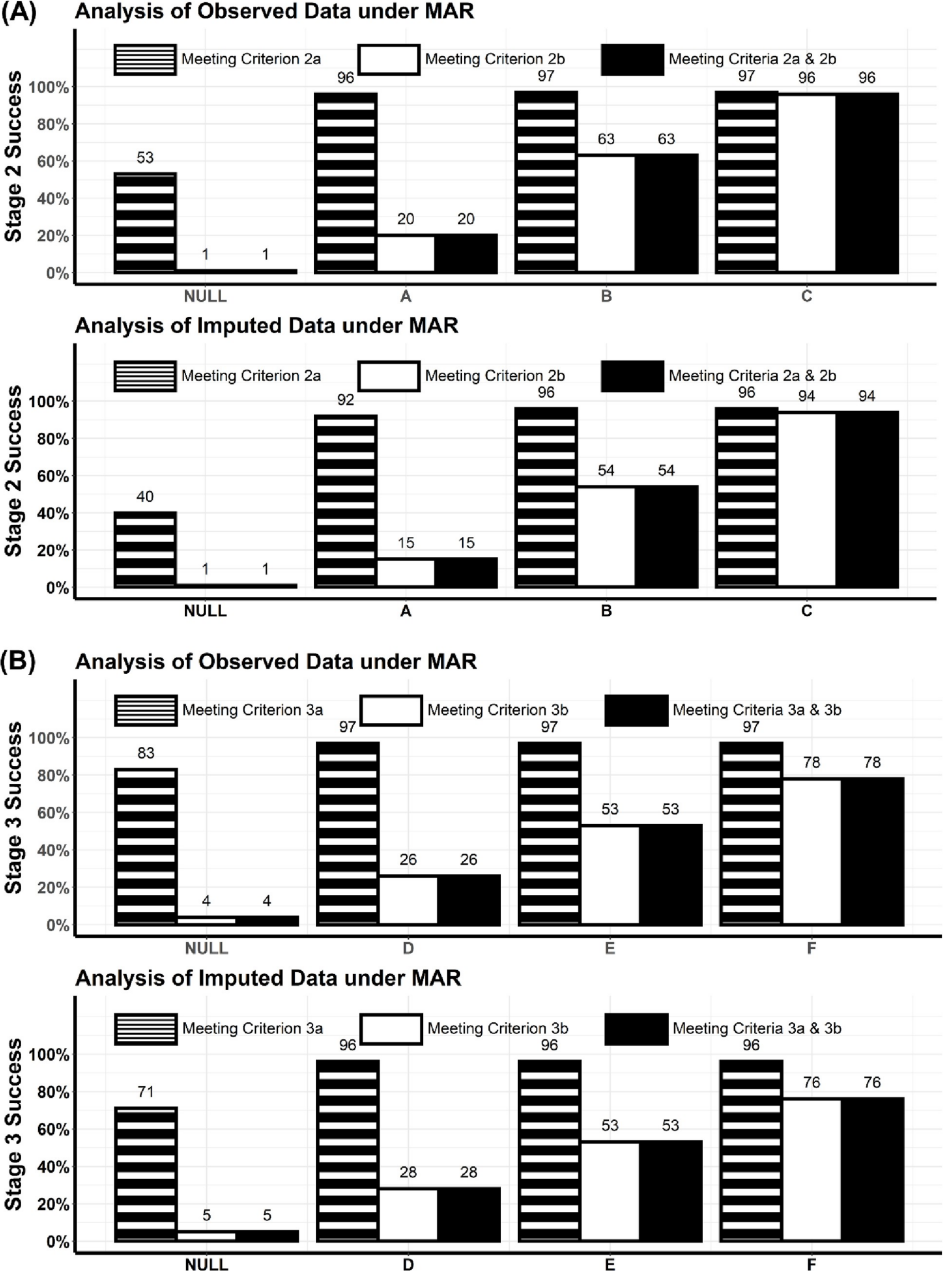
(A) Powers for demonstrating a Stage 2 effect by meeting success criterion 2a, or success criterion 2b, or both success criteria 2a & 2b under Stage 2 scenarios. (B) Powers for demonstrating a Stage 3 effect by meeting success criterion 3a, or success criterion 3b, or both success criteria 3a & 3b under Stage 3 scenarios. Note: Refer to Fig 2(B) for various effects under Stages 2 and 3 scenarios.

Since estimates tended to be biased under MNAR, we chose to present the powers for Stage 2 or 3 success under MAR only (Fig 3). Fig 3(A) shows the powers for meeting one success criterion (2a or 2b) or both success criteria under various Stage 2 scenarios. As noted above, type I error rates under the null scenario were largely inflated for meeting success criterion 2a while preserved at the nominal level for meeting success criterion 2b or both success criteria. The powers for meeting success criteria 2a were above 92%. The powers for meeting success criterion 2b/both criteria were 20%, 63%, and 96% from analysis of observed data under Stage 2 scenarios A, B, and C, respectively. Analysis of imputed data resulted in slightly less powers (i.e., 15%, 54%, and 94%, respectively), possibly due to slightly larger biases and standard error estimates after multiple imputation.

Fig 3(B) shows the powers for meeting one success criterion (3a or 3b) or both success criteria under various Stage 3 scenarios. We observed a similar pattern in type I error rates. The powers for meeting success criterion 3a were above 96%, and the powers for meeting success criterion 3b/both criteria were approximately 26%, 53%, and 78% under Stage 3 scenarios D, E, and F, respectively.

## Discussion

In our previous report [1], we introduced the framework of a three-stage design for stagewise evaluations of the efficacy of an AIT product. The proposed framework provides end-of-stage inferential statistics to inform clinical understanding of an investigative AIT over time. Whether the comparative results from end-of-stage analyses alone are adequate to support a Stage 2 or 3 effect is debatable, because end-of-stage difference between two groups may depend on the duration of Stage 2 or 3. Ideally, Stages 2 and 3 should be of sufficient duration such that the treatment effect is manifested during Stage 2, and a sustained effect can be captured at the end of Stage 3, respectively. However, if Stage 2 or 3 are curtailed for any reason, effect may be falsely claimed based on end-of-stage difference alone.

Here we introduce a trend analysis to evaluate the difference in progression curves of two groups over the entire stage. For example, if there exists a constant distance between the two progression curves (i.e., equivalent slopes), this will indicate that the end-of-stage benefit will persist, and particularly not diminishing, regardless of the duration of the stage. Since equivalence of slopes between the two groups over the period of Stage 2 or 3 indicates that the end-of-stage benefit does not diminish over time, trend analysis may provide more vigorous evidence for a Stage 2 or 3 effect than end point alone. Together, end-of-stage and trend analyses of Stage 2 data can solidify information on how time of introduction and length of treatment will impact the response to treatment over time to inform an optimal duration. Subsequently, joint results from end-of-stage and trend analyses of Stage 3 data will inform of the duration of benefit after treatment is withdrawn—i.e., whether there is a sustained effect.

Since multiple hypothesis testing are proposed across stages, discussions may be necessary with respect to multiplicity issues. We designed the continuation of the study in later stages to be dependent on a Stage 1 success, and thus, type I error control is in place via gate-keeping strategy from Stage 1 to Stages 2/3. Furthermore, since the two hypotheses in Stage 2 (2a and 2b) or Stage 3 (3a and 3b) are intersection-union testes, type I error rate is controlled at a conventional significance level (i.e., 0.05) for Stages 2 and 3 analyses, respectively (shown in our simulation results). We consider a controlled type I error rate for each of Stages 2 and 3 analyses adequate and acceptable because analyses of Stages 2 and 3 data are indented to provide information on the timing and duration of benefit on and off immunotherapy, respectively. In case where clinical decision/firm conclusions are to be made at the end of Stages 2 and 3, because of the chronological nature of the design resulting in hypothesis testing being conducted sequentially for the two stages, we consider it meaningful and acceptable to address the unique and equally important objective of each stage (one after another) at a conventional significant level as if they were separate studies.

There remains caveat to the study outcome and statistical approaches that we chose. A continuous outcome was repeatedly measured in our simulation study. For simplicity, a linear trend was assumed for Stages 2 and 3 such that the pattern of the effect over the entire stage could be evaluated by a slope ratio. However, the assumption of a linear trend may not be appropriate. For example, the symptoms of seasonal allergy are cyclical and depend upon pollen levels, which may be unpredictable and vary from year to year. When a linear assumption is implausible, other statistical measures such as area under the curve (AUC) may be appropriate to capture the disease burden during the entire stage. In addition, a non-parametric model may be an appropriate alternative to evaluate a shift manifesting treatment effect. Therefore, the type of model for trend analysis and the statistical measure for capturing the trend should be specific to the allergic disease under investigation. For example, the outcomes and models for food and environmental allergy may not be the same.

Furthermore, periodic assessments may be available so that the time course of the effect over the entire stage can be analyzed in the trend analysis. However, when the study outcome is not repeatedly measured, it may not be clear whether the comparative results from end-of-stage analyses alone sufficiently support the conclusion of a sustained effect.

Finally, these proposed analysis strategies are designed towards predicting trends of the benefit of different durations of treatment (i.e., treatment versus control in Stage 1) as well as persistence of that benefit after withdrawal of treatment. While these may point towards a binary decision as to whether the proposed treatment is beneficial, trends may also guide investigators toward study design choice such as length of each stage and clinically meaningful difference margins. These trends may also be useful for clinicians towards choosing optimal lengths of treatment and observation periods after withdrawal.
